# From Action to Cognition: Neural Reuse, Network Theory and the Emergence of Higher Cognitive Functions

**DOI:** 10.3390/brainsci11121652

**Published:** 2021-12-17

**Authors:** Radek Ptak, Naz Doganci, Alexia Bourgeois

**Affiliations:** 1 Division of Neurorehabilitation, University Hospitals Geneva, 1205 Geneva, Switzerland; 2 Laboratory of Cognitive Neurorehabilitation, Faculty of Medicine, University of Geneva, 1205 Geneva, Switzerland; naz.doganci@hcuge.ch (N.D.); alexia.bourgeois@unige.ch (A.B.)

**Keywords:** neural reuse, action emulation, motor planning, functional network, distributed knowledge, visual agnosia, lesion studies, mental rotation, embodiment

## Abstract

The aim of this article is to discuss the logic and assumptions behind the concept of neural reuse, to explore its biological advantages and to discuss the implications for the cognition of a brain that reuses existing circuits and resources. We first address the requirements that must be fulfilled for neural reuse to be a biologically plausible mechanism. Neural reuse theories generally take a developmental approach and model the brain as a dynamic system composed of highly flexible neural networks. They often argue against domain-specificity and for a distributed, embodied representation of knowledge, which sets them apart from modular theories of mental processes. We provide an example of reuse by proposing how a phylogenetically more modern mental capacity (mental rotation) may appear through the reuse and recombination of existing resources from an older capacity (motor planning). We conclude by putting arguments into context regarding functional modularity, embodied representation, and the current ontology of mental processes.

## 1. Introduction

**Figure 1:**
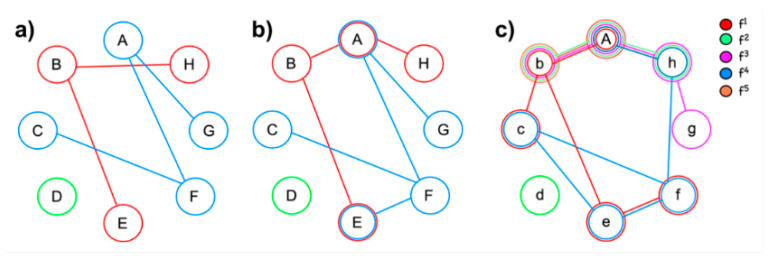
Schematic representation of neural reuse as compared to domain specificity and encapsulation. Letters stand for specific brain areas (or resources), while colors represent distinct functions (f^1^–f^5^). (**a**) Model of entirely separated processing, where each function recruits distinct areas, and no area is shared between functions. (**b**) Partial area/resource sharing (A and E are shared between two functions). (**c**) Strong area/resource sharing (A participates to all 5 functions, b to 3 different functions etc.).

The term *neural reuse* describes the capacity of the brain to adapt to changing demands by reutilizing some of its structures or resources in a new context (Figure 1). Though reuse is a form of plasticity, it should not be taken as implying just an adaptation or strengthening of specific brain circuits, such as when learning a new skill or increasing one’s vocabulary. Reuse has a much broader sense, as it refers to more fundamental changes taking place at the individual and, for some authors, the species level. Adaptations requiring neural reuse may be triggered by environmental pressures, leading to the reattribution of neural resources to alternative activities while remaining available for the original function. Alternatively, they may be driven by individual development, stimulating the use of skills and competencies when a child is confronted with a new problem. Neural reuse is thus a fundamentally adaptive feature of the brain. Its result will not be just a modification of the existent behavioral (or cognitive) repertoire, but the emergence of an entirely new capacity. Neural reuse ultimately results in a structural reorganization of brain circuits, but also in a new arrangement of computational operations.

This general overview anticipates a more detailed discussion of theoretical proposals taking different perspectives. The aim of this article is to discuss the logic and assumptions behind the concept of neural reuse, to explore its biological advantages and to demonstrate the benefits of reuse at the cognitive level. In particular, we will discuss developmental approaches [1], the concepts of embodied versus symbolic knowledge [2,3], the reshaping of cortical representations through the acquisition of new capacities [4] and how distributed processes facilitate neural reuse [5]. We will further consider how two seemingly opposite concepts that are relevant for neural reuse (localized vs. distributed representations) can be accommodated through interchanges between different methodological approaches. Further, we will elaborate on neural reuse by proposing how a mental capacity (mental rotation) may emerge through the recombination of existing resources. Finally, we conclude by discussing some open issues of neural reuse and address future challenges.

## 2. Local or Distributed: Two Views of the Structure-Function Relationship

The notion of neural reuse can be tracked down to controversies regarding brain–behavior relationships, in particular the questions whether knowledge is distributed and whether brain regions may specialize for a particular function. While early observations by Broca [6], Lissauer [7], Bálint [8] or Gerstmann [9] and others suggested a high degree of specialization of distinct brain areas for language, visual perception, arithmetic or even recognition of body parts, later authors emphasized the difficulty to find the precise location of representations in specialized parts of the brain. Thus, after reviewing results from other studies and himself performing lesion studies on various parts of the rat brain, Lashley [10] concluded that brain function depended on volume, not the precise location of the removed cortex. Lashley noted: ‘There is evidence of mutual dependence of parts in which the specialization of structures seems less important than the mere mass of functional tissue. There are indications that (…) the subordinate parts are all equally capable of performing the functions of the whole’ ([11], p. 34). Based on his data, he proposed the concept of *equipotentiality*, which reflects the capacity of the intact cortex to take over any function from an injured brain region. Equipotentiality is not only incompatible with local representations, but also with other characteristics of a strongly modular view, such as domain-specificity and the encapsulation of computational processes. Other important thinkers were Geschwind, who emphasized the importance of white-matter connections for brain functions and thus a more dynamic view of the brain [12,13], and Edelman [14], who disputed the ‘unidirectional’ information processing view of brain function underlying the information processing approach, according to which external inputs are fixed and devoid of variation. Edelman proposed that local anatomy at the level of cell groups varies across individuals, as it is subject to environmental variation present during individual development. He also introduced the concept of *reentry*, a continuous signal exchange between higher-order and lower-order levels of a neural hierarchy. His most important concept for the current review is *degeneracy*, a term borrowed from biology to describe the variable relationship between function and structure. When applied to brain function, degeneracy reflects the capacity of structurally dissimilar brain areas to carry out similar functions under some conditions, but different functions under other conditions. As we will see, this is a fundamental premise of neural reuse theories. Though Edelman’s ideas have mainly been used to describe behavior of cell populations, they were later applied to psychophysical phenomena [15] and brain circuits or networks [16,17]. Another important development led to the emphasis on distributed processing as an alternative to the localized and static input–output relationships proposed by cognitivists [18,19,20,21,22].

The notions of neural reuse and distributed processing have early met their adversaries, however. For example, after examining some of Lashley’s data, Thomas [23] concluded that parietal damage had significant effects on maze performance of rats irrespective of the volume of damage. This was a statistical argument against mass action and in favor of (at least broadly) localized representations. From 1950 on, lesion studies of individual patients continued to provide evidence for specific impairments following focal brain damage, such as amnesia [24,25], prosopagnosia [26], simultanagnosia [27] or finger recognition impairment [28], and studies on disconnection [29] further provided evidence for functional specialization across the hemispheres. During the same period, the logic and methodology behind the component analysis of cognitive processes was attuned [30,31], and analyses of processing stages such as the subtractive method were improved [32] (see also [33]). Concepts of ‘modular function’ and ‘encapsulation’ received a rational basis [34,35], and provided the reason why cognitive neuroscience was influenced by the ‘localist’ view well into the era of functional imaging. As a consequence, some early findings of seemingly ‘local’ representations were interpreted as reflecting a cortical module, that is, a highly specialized functional cortical area [36,37,38,39]. How strong and relevant the contribution of lesion studies was, and continues to be, is exemplified by the simple fact that without them we would not know about syndromes such as prosopagnosia, spatial neglect or even amnesia. As we will show, however, at least for some theorists, neural reuse is entirely incompatible with a localist and modular organization of the mind.

## 3. Theories of Neural Reuse

The concept of neural reuse is not conceivable without several underlying assumptions. First, it involves an evolutionary perspective, where neural changes are triggered by a need for adaptation to changing environments. Within an evolutionary perspective, neural reuse is a form of exaptation, that is, the adaptation of a specific trait to serve a new function while also maintaining its original function [40]. Here, we will address such evolutionary aspects of neural reuse only superficially, since they are less known and more subject to conjecture than ontogenetic aspects. Of much greater relevance for the topics discussed in this paper is the second point: reuse is expressed during development and maturation and is therefore dependent on neural and cognitive plasticity. The type of plasticity that is at play contributes to a relatively consequential reshaping of the cognitive anatomy of the brain and operates at the level of brain circuits and networks. It is therefore more than merely a fine attunement and optimization of already existing circuits. Importantly, it is involved in combining mental operations that are based in motor and sensory cortices with each other and with higher-order processes grounded in the associative cortex. A third important assumption says that neural reuse is the manifestation of the reorganization of a complex system, which must respect principles of neural economy and optimization. This assumption reflects the biological constraints that impose a barrier on the brain’s energy consumption. According to the fourth premise, reuse must be subject to internal competition and cooperation, as any change of function and its underlying neural implementation cannot go without accompanying effects on neighboring structures or related functions. However, reuse also requires neural synergy, since a resource must be inserted into a new ensemble to facilitate the emergence of a new computational unit. Finally, reuse reflects a significant amount of redundancy of neural systems, rendering them more resistant to damage, yet more flexible and responsive to external pressures.

These assumptions are present in several theoretical currents that assume some form of reuse. One such current assembles developmental scientists who conceive the emergence of mental structure within what has been termed a neuroconstructivist framework. For example, according to Karmiloff-Smith [1], cognitive processes are initially usable for different types of inputs and gradually (through individual development) become more specialized for a particular domain. Thus, domain-specific representations are not genetically predetermined, but emerge through changing interactions with the environment. This leaves room for different developmental trajectories, during which disorders of development may profoundly affect the cognitive architecture. Hence, seemingly intact performance in developmental disorders does not indicate the preservation of specific modules, but may be achieved through an alternative cognitive organization. Neuroconstructivists criticize the nativist view of a static, encapsulated and hard-wired mental architecture, and instead emphasize the flexible representational capacities of the brain that are shaped through its interaction with the environment [41].

Another group of theories are based on the idea that the same cognitive processes are involved when we perform a particular behavior, or just imagine performing this behavior. For example, imagining (i.e., simulating) the grasping of an object activates the same neural structures, motor processes and cognitive representations as actually grasping the object. While some authors have applied the simulation idea specifically to motor cognition [42] others have gone further to explain the understanding of other people’s actions, intentions, emotions or, more generally, internal states through simulation [43,44]. Several authors have designated mirror neurons (specialized cells of monkey premotor cortex, which are active when a monkey performs an action or merely observes someone else performing the action) as the neural basis for simulation [45,46,47]. A closely related current is based on the idea that sensory or motor cortices retain a flexibility to serve other than their original functions. Gallese and Lakoff [3] propose ‘neural exploitation*—*the adaptation of sensory-motor brain mechanisms to serve new roles in reason and language’ (p. 456) as a fundamental characteristic of neural implementations of cognitive processes. These authors argue that a concept such as ‘grasp’ only becomes meaningful for a mind that knows how to grasp, has perceived and can imagine grasping. Because knowledge structures depend on sensory and action structures (i.e., are embodied), concepts cannot be symbolic and referential. Similar ideas about the expansion of function have been put forward to explain functions other than language, such as attention [48]. For example, in their *premotor theory*, Rizzolatti et al. [49] proposed that spatial attention has appeared by splitting from the evolutionary older saccade programming system. Corbetta et al. [50] in turn posit that the spatial reorienting system of attention has been exploited for perspective taking, and thus to understand other people’s intentions.

The *neuronal recycling* theory aims to explain cognitive structure by giving particular weight to interactions between genetic and cultural influences [4]. The theory posits that the domain-specificity of the cortex is in part genetically predetermined, in that an organization of cortical areas into motor or sensory maps is subject to inherited anatomical constraints (such as the cortical projections of the optic radiations). Culturally transmitted abilities must therefore find a neuronal representation in an area that has the required computational characteristics and is sufficiently plastic to accommodate the new function. The theory focuses on abilities that have appeared within the last couple of thousand years and can therefore not have a genetic basis, such as reading, writing and mathematics. For example, illiterate participants exhibit symmetrical activity in inferior-temporal cortex to faces, while literate subjects show a bias towards stronger responses to faces in the right hemisphere [51]. By testing participants with different degrees of literacy, the authors could demonstrate that the acquisition of reading gradually encroached upon the face-sensitive areas in the left hemisphere. Thus, in agreement with neuronal recycling theory, a culturally acquired ability (reading) reshapes cortical maps by competing with a genetically transmitted ability (face processing).

Possibly the most radical theory of neural reuse is the *massive redeployment* hypothesis of Anderson [5,40,52]. Anderson starts with an argument of economy: given the excessive energy consumption of the brain, it is more economical to reuse existing circuits than to develop new ones. Therefore, we should ‘expect a typical brain region to support numerous cognitive functions in diverse task categories’ ([5], p. 246). Importantly, he expects that phylogenetically older areas should have been reused more often during evolution and therefore participate to various cognitive functions, and more recent cognitive functions should involve more scattered brain areas. Anderson cites evidence from functional imaging showing that brain areas are activated by a variety of different tasks [53,54], and that language (presumably a recent acquisition in human history) activates more distributed and scattered brain regions than perception or attention [40]. He finds it logically invalid to say that the brain is composed of functionally independent and completely separable parts (i.e., modules). Consequently, he disapproves of the decompositional approach of cognitive neuroscience (to analyze distinct parts by studying functional dissociations) and advocates instead for a network approach: to seek for higher-order patterns in complex networks and relate them to behavior. However, as we will see in the next section, even network models cannot escape the discussion about the structure-function relationship, and strong data suggest that network structures are not incompatible with some form of functional modularity.

In sum, neural reuse theories distinguish themselves by emphasizing the interaction between inherited characteristics of brain circuits and environmental (or cultural) pressures requiring a new cognitive function to be accommodated within existing structures. Most of them assume some form of exaptation, that is, the reutilization of a phylogenetically old trait or structure in a different context. They argue against a localist view of brain function, and some of them put into perspective the assumption that cognitive architectures are universal.

## 4. Functional Networks Provide the Necessary Conditions for Neural Reuse

**Figure 2:**
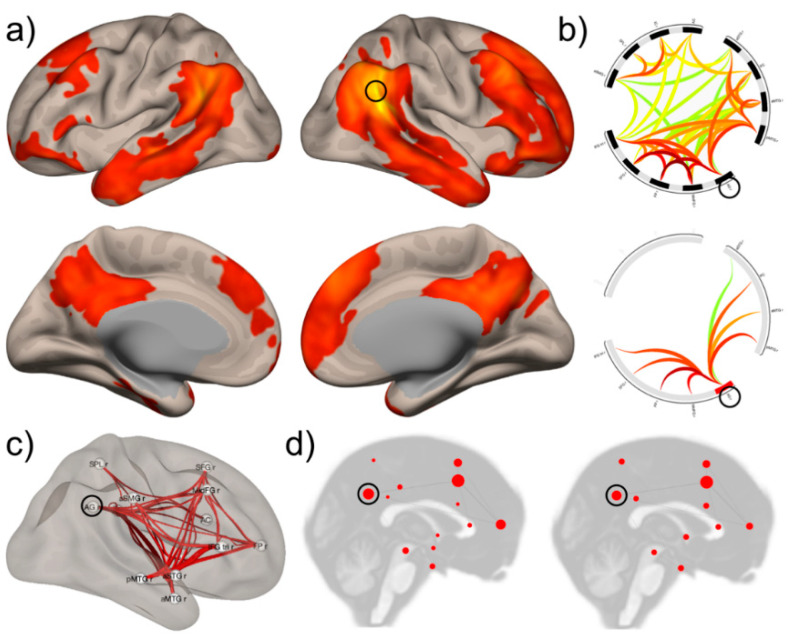
Measures and representations of functional connectivity. The small black circle represents an area/region of interest considered a cortical ‘hub’ (right angular gyrus, rAG). (**a**) Seed-to-voxel functional connectivity identifies within- and between-hemisphere connectivity of the rAG with frontal, temporal and medial associative cortex of both hemispheres. (**b**) Region-of-interest connectome involving 13 right-hemispheric hubs (upper ring) and only connections involving the rAG (lower ring). (**c**) Another representation of the same data as shown in (**b**). (**d**) Graph-analytic network representation, where circle size is proportional to graph theory measures of degree (left) and betweenness (right). Data from [79].

The contrast between a localist versus distributed view of brain function has been accentuated when functional imaging shifted from focusing on brain activity to the examination of functional networks. We believe that the analysis of functional networks provides a solution to this controversy by identifying different network characteristics and important network components [55,56,57,58,59]. The logic of network analysis can be summarized in the following propositions (see Figure 2):

1. The brain is organized into large-scale, highly interactive networks, whereby there are many more short-range (local) than long-range connections [56,60,61]. This architecture maximizes efficiency while minimizing energy consumption [62].
2. Networks consist of nodes and connections, whereby some nodes (‘hubs’) are of greater importance than others and some connections are stronger than others [63,64,65]. The intrinsic organization of networks enables processing information along a continuum of encapsulation: they may adopt a centralized and modular state (segregation) or become more penetrable to influences from other networks (integration; [66]). Integration and segregation of information processing are thus expressions of the current state, not a fixed feature of networks. Note that the term ‘modular’, when applied to networks, relates to the formation of subgroups of nodes forming a community within a network [67].
3. A network structure appears at rest or during activity. The high degree of energy consumption during ‘rest’ indicates that a large quantity of information processing is intrinsic and occurs without external stimulation [68].
4. Though the spatial and temporal stability of networks is under debate [69,70], at least some studies using task-based connectivity suggest temporal changes of node weights and network topology over time [71,72,73]. There is also evidence of interindividual variability, yet intraindividual stability of networks [69]. This corresponds to one of Edelman’s requirements for theories of brain function, namely to explain ‘how both perceptual and conceptual categorization can arise as a result of selection upon preexisting variance in structure and function of the nervous system’ ([14], p. 115).
5. Focal lesions have local as well as distant effects on network function [74,75,76,77]. Distant effects modify intrahemispheric networks or cross-hemispheric connections and may manifest in increased or decreased activity of distant brain regions [76,78].

These propositions depict a much more dynamic view of brain function than what classic lesion studies, but also early studies using functional brain mapping, have implied. Information processing is the result of segregation and integration, whereby information may at times be encapsulated, or be exchanged across networks. Modularity and distributed processing are thus relative—not absolute—characteristics of such a system. This does not mean that a network cannot have a fixed architecture, but rather that the strength of connections in a functional network may vary despite a stable network structure. Even network function can be relatively fixed, as long as some basic operations remain available for alternative functions. Thus, a given network may be preferentially involved in a particular function (e.g., motor programming and execution), yet some of its functional and structural components may be reused by other networks to generate other functions. Additionally, developmental studies indicate that motor and sensory networks appear earlier during brain maturation than networks associated with functions such as working memory, attention or cognitive flexibility [80,81,82]. The level of functional specialization of these ‘higher-order’ networks is much less fixed, and different network configurations may be associated with distinct functions [53,58]. Thus, networks may ‘borrow’ sub-components for particular tasks and may contribute to what is not necessarily their primary function. This conclusion applies particularly to higher cognitive functions, which may emerge from the recombination (or aggregation) of several low-level operations [83].

In sum, brain networks are characterized by a high degree of functional diversity, which provides a building stone for a highly interactive brain and neural reuse. Is this conclusion incompatible with the main assumption of lesion studies, namely that if a lesion produces a deficit in function X, then the damaged area must somehow be *necessary* for that function? We do not think so. First, the major contribution of lesion studies has been the precise analysis of cognitive architecture through the identification of functional sub-components, not the identification of specialized brain areas. These studies deal with functional associations and dissociations, and these—not the anatomo-functional relationships—constitute the building blocks for theoretical reasoning in cognitive neuropsychology [84,85,86]. Second, many authors did not really bother about the precise lesion location or even study patients with relatively diffuse brain damage [87,88,89]. This approach is perfectly compatible with Fodor’s description of a modular function [34], as he merely argued that modules have a neurological substrate, but did not posit that this substrate must be in a circumscribed area of the brain [90]. Indeed, Fodor mainly argued for *functional*, not anatomical modularity. Third, what is perceived as a weak point of lesion studies—that damage to specific brain regions may have different effects across individual patients [16]—has been explicitly acknowledged in single-case methodology [30,31,91]. The main assumption here is that cognitive architectures apply universally to all individuals (for example, we all read by using the same functional components), not that the neural implementations of these architectures must be universal. Finally, while the lesion method undoubtedly has many problems [92,93] it is questionable whether another method may provide a strong criterion against which the validity of a specific brain region being involved in a specific function can be verified. For example, functional MRI lacks specificity (it often reveals activations in areas that are not essential for a given task) and temporal resolution. It is therefore not obvious to identify a region’s importance, or its contribution to a specific cognitive process, from brain activity alone [54]. In conclusion, to understand brain–cognition relationships, we have to confront findings from neuroimaging and lesion studies against each other, adapt our paradigms and integrate new observations in an iterative process [92,94]. Proceeding in this way may best benefit from the advantages of each method. The next section will exemplify how cognitive models may benefit from such an integration of lesion and functional imaging data.

## 5. Integrating Neural Data and Behavior: The Case of Visual Object Processing

The lesion approach struggles with the concept of distributed functions, and findings of impairments following focal damage are often considered to be incompatible with distributed processing. This was not so much of a problem as long as data on the distributed nature of information was lacking, but became a reason of controversy with the advent of functional imaging. We next discuss how functional imaging may complement lesion studies to accommodate a rare neuropsychological syndrome—visual object agnosia—with a distributed view of perceptual processing.

Since its original description by Lissauer [7], visual agnosia has been seen as a disorder affecting distinct ‘stages’ of processing. Lissauer distinguished between ‘apperception’ (establishment of a conscious percept) and ‘association’ (access to the meaning of the percept), while later writers trained in the Gestalt tradition particularly focused on different grouping processes [95]. Later evidence motivated more elaborate models, consisting of three or more computational levels thought to be arranged hierarchically, where operations on visual primitives always precede higher-level analysis such as shape integration and elaboration of a 3-dimensional representation [96,97,98,99,100]. This short overview exemplifies the typical development of concepts and increasingly complex models when more data from behavioral studies on healthy participants or brain injured patients become available. In what follows, we focus on visual object agnosia, a term that refers to patients who are capable of analyzing perceptual characteristics of an image, yet fail to ascribe meaning to it [101]. These patients show preserved elementary perceptual processes, are able to produce adequate copies of objects and pictures and can make detailed perceptual judgments or differentiate between two closely similar objects [102,103]. By contrast, they fail when asked to provide the name of a shown object, report its semantic characteristics or communicate an associated object [104,105].

While there are differences between individual patients, it is well established that patients with visual object agnosia may exhibit very selective deficits [106,107,108]. For example, they may show no lexical or semantic impairments when their knowledge is tested verbally, naming objects correctly when given a definition or when allowed to touch the item. They also easily perform associative tasks on names of objects, even though they fail the same task when shown pictures of the same items. Based on these observations, Teuber [109] famously characterized visual object agnosia as a disorder in which a percept is stripped of its meaning.

While the behavioral features of visual object agnosia are relatively well accepted, the apparent incompatibility of neuropsychological and functional imaging findings is perplexing. Approximately two thirds of all patients have left inferior temporo-occipital damage, while the rest have either bilateral or unilateral right damage [110,111]. Thus, lesion studies suggest a dominance of the left hemisphere for the type of processing that is required for a perceptual representation to access semantics. However, not all patients with an inferior temporo-occipital lesion develop visual object agnosia; in fact, the disorder is quite uncommon [112]. A further problem is that many functional imaging studies of healthy participants report bilaterally distributed activations across large portions of the occipito-temporal cortex [113,114]. A particularly effective technique is to compare activations to entire objects with randomly scrambled object parts. This approach reliably generates activations of lateral occipital cortex (LOC; [115,116,117]) in both hemispheres. Thus, there seems to be a gap between clinical findings suggesting left-hemispheric dominance and functional imaging reporting distributed activations [118]. However, this gap can be closed when one considers findings from a few functional imaging studies examining patients with agnosia [119,120,121]. For example, the intact-scrambled comparison was performed with a patient who had developed visual object agnosia following a left occipito-temporal lesion sparing primary visual cortex [122]. When the patient was shown faces (a relatively preserved category), he showed no activation of his left (damaged) associative visual cortex, but significant activation of preserved right occipito-temporal regions. However, when watching intact versus scrambled objects, the patient exhibited no significant activation, even though the same contrast reliably activated the bilateral LOC in all control subjects. Thus, unilateral damage resulted in a bilateral deficit of brain activity, and this was specific to the visual category for which the patient had a selective deficit. In addition, a later connectivity study showed that although functional, the right-hemispheric LOC of the patient was functionally disconnected from his left hemisphere [123].

These findings provide a viable solution of the distributed-localized controversy of visual object perception: for a specific stimulus category focal damage may have global effects on the visual system. This leaves open the possibility of a relative dominance, where object processing is generally distributed, yet individuals show varying degrees of lateralization. Such an explanation is compatible with the principles of network science, in particular the presence of network hubs that receive high numbers of connections and thus have privileged access to information. Damage to hub regions (such as the left LOC in our patient) may therefore have widespread effects across the entire network. Beyond the immediate relevance for models of object processing, the conclusion to keep in mind from these findings is that the interaction between lesion studies and functional imaging may accommodate both localist and distributed views of brain function.

## 6. A Motor Process for a Cognitive Function: The Emulation Theory of Mental Rotation

The aim of this section is to present a concrete example of neural reuse by showing how mental rotation may emerge as a combination of core functions relying on motor and perceptual systems. Our theory is based on Grush’s emulation theory of mental representation [124]. Grush argues that action control requires anticipation of the effects an action will have on the environment, and that these effects can be computed when a copy of the intended action is processed through a specific control device, which he terms the emulator. The emulator processes the copy in a way similar as the effector would and feeds its output back to the control device that has produced the action. According to Grush, the emulator adapts and filters noise inherent in the copy of the signal it receives by using a Kalman filter (a device that estimates a variable or ‘model’ by using repeated measurements, thus optimally controlling for noise and measurement error). He argues that such a device can produce adequate models of a motor plan and can be adapted to produce motor imagery. Conditions for the latter are that the emulator should be disconnected from real sensory information (putting it in a mode which only considers internally generated information), and the motor command should not affect the body. This produces a system that disregards real sensory information and applies motor plans on generated images of the body.

We here propose a very similar model for mental rotation. Mental rotation is the cognitive process that allows observers to make explicit judgments about stimuli that have been rotated away from an intrinsic axis. The primary result of mental rotation experiments is a systematic (often linear) relationship between the rotational angle and a reference position of the stimulus [125,126,127]. This relationship can be expressed with a regression function whose slope indicates the relative speed of the mental transformation process. While possible differences exist in mental rotation of different stimulus types (which we will discuss later), the nearly linear relationship is a stable characteristic. Mental rotation therefore provides a prime argument for an analogical process, suggesting that mental representations are modal rather than symbolic.

How could a cognitive function such as mental rotation emerge from pre-existing neural and cognitive architecture? First, we may assume that mental rotation relies on several cognitive building blocks, amongst them perception (the target has to be identified), mental imagery (an image has to be generated), working memory (the image has to be maintained) and decision processes (the transformation process has to be stopped and a decision has to be reached). Our focus here is on the mental transformation process itself, which is not covered by either of the latter functions. We propose that an additional building block is ‘borrowed’ from the motor system to perform the transformation operation.

**Figure 3:**
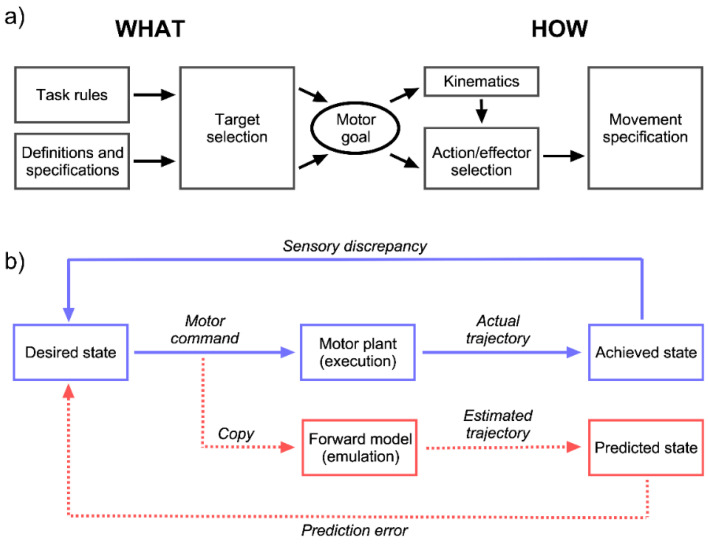
Models of motor planning. (**a**) Flow-model differentiating What- and How-processes, where motor planning follows a temporal flow, and What-processes are supposed to occur in parietal cortex, while How-processes depend more on premotor cortex (modified from [128]). (**b**) A forward, or predictive, model of motor planning, where feedforward processes proceed from left to right and feedback processes use comparators to evaluate the discrepancy between achieved/predicted vs. desired outcome of a motor plan (modified from [134]).

**Figure 4:**
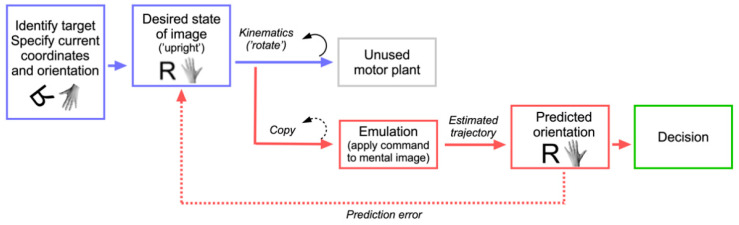
The emulation theory of mental rotation. Processes drawn in blue have mainly to do with stimulus identification, analysis of spatial task parameters and definition of a desired outcome. Red processes relate to the emulation process that has been ‘borrowed’ (reused) from the motor system. Abstract kinematics are defined at the level of ‘command’, and a copy of these is applied to the mental image. The decision process is shown in green.

In order to understand which motor process could be involved, we have to examine the cognitive architecture of motor planning. Computational models of motor planning distinguish between processes related to ‘what’ the object of the action is (localization strategies, stimulus selection, application of task rules) and ‘how’ the action has to be performed (computation of kinematics, selection of the effector, movement specification; see Figure 3) [128,129,130,131]. It is important to note that most of these processes occur offline and—except for the very last stages—could in principle take place without the action being executed. The motor system is therefore prepared for mental rehearsal, or simulation. This is made possible because control is not only achieved through feedback, but also feedforward mechanisms that allow adjusting or interrupting motor plans quickly upon changing target conditions [129,132,133,134]. If control was only based on feedback mechanisms, it would not be able to predict adequately sudden changes in target parameters, since it would constantly remain in a reactive instead of a proactive mode. A forward model compares the predicted outcome of an action with the desired outcome [135]. As mentioned when we introduced Grush’s emulation theory, it is created through a copy of the motor commands at an abstract stage where motor kinematics are computed [134]. We propose that the component of motor planning which is best prepared for rehearsal must be located after the what-processes are concluded, but early in the stream of how-processes. The reason is that it should be located before the effector has been selected and the action has been initiated [136]. In analogy to Grush, we propose to call this abstract stage motor emulation, and our theory the *emulation theory of mental rotation*. Figure 4 shows a representation of our model in a box-and-arrows diagram.

It is challenging to imagine the type of representations that are elaborated at the emulation stage. They should contain the action plan, formulated in terms of its general spatial and temporal characteristics, but not a specification of the effector. A simple way is to imagine the movement that is associated with writing the letter S. The abstract plan defines a starting point, then a counterclockwise semi-circular movement, followed by a similar movement in the clockwise direction. Importantly, being independent of the effector, this abstract motor plan could be performed with the dominant or non-dominant hand, individual fingers, each of the two feet or even the head. In each case the basic movement characteristics would be the same, even though movement execution would differ in terms of size, speed or smoothness. Thus, the kinematic plan has all characteristics that are required for mental rotation: a dynamic process defining movement onset, direction, speed and offset. The central assertion of our theory is therefore that mental rotation emulates the motor kinematics that would be involved if a limb was actually physically rotated. Before discussing the implications of our propositions, we would like to add an important specification. We previously suggested that the reused motor process should not be too close to effector specification, in order to contain relatively pure action kinematics. This is true for the mental rotation of non-bodily stimuli, while evidence suggests that pictures of body parts may automatically activate the depicted effector. As a result, mental rotation of body parts will be more effector-dependent than the rotation of non-bodily stimuli. In sum, the theory makes the following predictions:

1. Development of mental rotation ability depends on the maturation of the motor system and only becomes available once the child is capable of replaying actions mentally.
2. Mental rotation and motor execution produce comparable patterns of performance. For example, the effect of rotational angle in mental rotation of hands is proportional to its effect when subjects actually perform hand rotations. Hand positions that are difficult or impossible to imitate produce particularly increased reaction times.
3. An overlap exists between neural structures involved in motor planning and mental rotation.
4. Motor planning and mental rotation use similar resources, resulting in interference when both are performed simultaneously.
5. Mental rotation of objects relies on the same kinematic plan as the rotation of body parts, except for the involvement of an effector component in the latter.

Unfortunately, it is not easy to collect strong evidence for all five points; in particular, few data are available for the first and last point. As regards development, at 2–3 months infants produce their first directed movements (such as grasping) that, though uncoordinated, are explicitly aimed at an object [43]. Some studies indicate that directed actions might already be available at birth, but the movement is so jerky and irregular that it is difficult to tease apart the effects of inaccurate target specification and faulty execution [137]. At seven months infants adjust grasping movements (e.g., hand aperture) correctly, and they do it even when the object is not visible [138]. This finding suggests that they use a mental representation of object size to adapt their grasp. While infants start to rotate objects at 22 months to fit them in appropriate holes, they can actively participate in mental rotation experiments only when they are 4–5 years old [139,140]. Finally, the mental rotation abilities of 5–6 year-old children correlate with their motor competency. These figures thus support the first prediction of the theory.

The second point finds good support from several studies on the laterality judgment of body parts. For example, Parsons [127] showed that for subjects holding their hands palm-down a rotation judgment on hands that matched this position was easier than for hands shown palm-up. In several other experiments, he showed that the time to decision depended on the extent of biomechanical constraints imposed on a movement that would have been necessary to produce the depicted orientation. These are strong and robust findings that have been confirmed by several other studies [126,141,142]. A more recent study found that the initial position of the participants’ arms (extended vs. flexed) influenced their hand laterality judgments [143]. These studies thus reveal a direct relationship between the trajectory through which the subject’s hand would have to be moved and oriented to match the depicted hand and the mental transformation that the subject performed during the task.

Regarding the third prediction, extensive functional imaging evidence shows overlapping activations in tasks related to motor planning and mental rotation. Unfortunately, many studies examined motor imagery as a measure of motor planning, and this often includes limb laterality judgment, which is itself a mental rotation task. Comparisons between motor imagery and action execution identified common activations in the primary motor and somatosensory cortex, as well as the ventral and dorsal premotor cortex, including the supplementary motor area (SMA; [144,145,146]). The limb laterality judgment task (i.e., identifying a body-part as belonging to the left or right side of the body) shows stronger activation of the superior parietal lobe (SPL) and dorsal premotor cortex, while kinesthetic imagery activates the ventral premotor cortex [147]. Several meta-analyses of mental rotation also revealed bilateral activations of the dorsal parietal and premotor cortex [148,149,150]. A closer look at studies comparing the mental rotation of body parts and objects directly suggests some differences, with body parts generally activating more the premotor cortex than objects, while the latter more consistently activate the SPL [151,152,153,154,155]. Despite this difference, when each condition is compared with the baseline, robust bilateral premotor and parietal activations are revealed whether mental rotation involves bodily or non-bodily stimuli [151]. However, though the functional imaging literature is rich on the topic of mental rotation and motor planning, it is often difficult to disentangle cognitive processes that are specifically related to kinematic plans and the transformation component necessary for mental rotation. For this reason, though the overlapping activations suggest recruitment of similar neural resources, a possible confound is that task-related decision processes, visual imagery or effector selection affected previous findings.

While functional imaging generally supports the bilateral involvement of the dorsal frontoparietal cortex in mental rotation, early lesion studies have suggested a specialization of the right posterior cortex [156,157]. More recent studies have expanded this observation by reporting that left anterior damage impairs the mental rotation of body parts or when subjects use a manual strategy, while right hemisphere damage impairs performance with objects or when participants use a visual strategy [158,159,160]. Unfortunately, the number of participants with damage to specific brain areas was low in all these studies. Finally, a study examined the effects of the inhibitory stimulation of the dorsal premotor cortex, an area that is crucially involved in motor planning [161]. The authors found impaired mental rotation of objects, and to a lesser degree hands, after premotor inhibition. Thus, though the evidence from lesion or inhibition studies is relatively modest, they support the conclusion that brain areas which crucially contribute to spatial processing and motor planning (parietal and dorsal frontal cortex) are also involved in mental rotation.

The fourth prediction of the emulation theory is that motor planning and mental rotation use shared cognitive resources. This prediction is supported by the observation that manual and motor rotation times show nearly indistinguishable patterns when all other task conditions are equal [162]. More importantly, when manual movements and mental rotation are performed simultaneously, interference is observed for movements executed along discordant directions [162,163]. The locus of interference has been identified at the level of movement planning [164], which is compatible with the prediction of the emulation theory. Another line of supporting data has been obtained with amputees or patients with chronic pain syndromes. One study found that upper limb amputees are slower than healthy participants when performing hand mental rotation [165], particularly if amputation concerned their dominant hand. More interestingly, patients with severe arm or shoulder pain were impaired both in terms of speed and accuracy in hand laterality judgment when the depicted hand was shown at large rotational angles [142]. This finding suggests that even though no actual arm movement was involved, patients adopted a strategy of mentally rotating their arm to find a match with the target hand. The link between motor planning and mental rotation appears to be so strong that even mental rotation hurts!

The final point predicted by the theory is that the mental rotation of non-bodily and bodily stimuli should only differ regarding the involvement of effector selection in the latter. This point is hard to prove since it is difficult to disentangle effector selection from the definition of kinematic plans. Though functional imaging studies show that bodily stimuli activate the premotor cortex and some parietal regions more than non-bodily stimuli, both stimulus types overwhelmingly share frontoparietal activations [155]. However, it is possible that functional MRI simply lacks the temporal resolution necessary to reveal specific activations due to kinematic planning. Using EEG, a recent study found that mental rotation is associated with a sequence of electrophysiological processes, suggesting several stages of processing [166]. The study found that while early processes (<150 ms) are characterized by pure effects of stimulus type (which is compatible with perceptual analysis before the mental transformation starts), later processes (>300 ms) reflect the rotational component irrespective of stimulus type [167,168] and are associated with activation of the right parietal lobe. This finding of common neural activation during the critical period when the mental transformation takes place supports a shared cognitive process for bodily and non-bodily stimuli. However, though this partly supports the prediction of the theory, more evidence is needed to distinguish the temporal stages of effector selection from the specification of kinematics.

## 7. Conclusions, Limitations and Open Questions

The aim of this article was to discuss the properties and advantages of neural reuse for theories of cognitive function. While we have focused on mental rotation, many other examples could be mentioned that support the reuse of phylogenetically older neural areas by more recent functions. For example, according to some authors, conceptual representations are rooted within, or at least sustained by, brain areas originally involved in motor and sensory functions [2,3]. There is also evidence that spatial representations in the parietal cortex have been reused for number cognition [169], or that a part of the spatial reorienting system is involved in social perspective taking [50]. Yet another example of neural reuse is the interaction between emotion and action control [170,171], as emotional stimuli may affect motor cortex excitability [172]. Finally, some studies indicate that the human episodic memory system emerged from a spatial orienting system within the medial temporal lobes [173]. This is only a short and non-exhaustive list of how higher—and often culturally more modern—functions rely on original neural resources.

Though there are strong arguments for neural reuse, the concept also has several shortcomings, which make some of our conclusions provisional. One point concerns the temporal scale at which reuse can affect functional reorganization. As we have pointed out previously, some theorists consider reuse to be a fundamental organizational principle that is effective at the species level, while others focus on more individual, developmental effects. In addition, network measures suggest even faster intraindividual adaptations that might be observed across different tasks. Applying the term neural reuse to all these phenomena presents the risk that it be considered a one-for-all principle that explains any plastic change in the brain. Another problem is that it is difficult to identify the driving force behind reuse, since it might be spontaneous, genetically determined or driven by functional demands [174]. A further point is that there must be some biological and functional constraints on neural reuse that have not yet been fully understood. For example, each of the above examples of neural reuse reflect systems that have high functional similarity (e.g., magnitude representation is reused for number representation; spatial reorienting is reused for spatial perspective taking, etc.). The precise boundaries that constrain the borrowing of sub-functions across functional domains must therefore further be determined.

By focusing on mental rotation, we were much less radical and courageous than other theorists, as even strong critics of embodiment concede that some form of embodiment may underlie mental rotation [175]. Yet, the neural reuse framework is only in its beginnings, and several serious questions remain to be answered before it becomes widely acceptable. One of these questions concerns ontological aspects of cognitive functions. Cognitive science utilizes terms to describe functions that are derived from concepts which have their roots in different disciplines (psychology, philosophy and linguistics), are relevant to parts of folk psychology or simply relate to subjective constructs [176,177,178,179]. The scientific taxonomy used to describe functions of networks or specific processes assigned to network nodes does not easily fit on such traditional definitions of function. A network configuration relates more to computational outputs, data visualization and cognitive processes that are possibly common to different tasks. We currently lack a vocabulary that would adequately characterize ‘functions’ in the sense of a network configuration [180]. The dilemma becomes visible in the equivocal names that are given to certain networks (e.g., frontoparietal, control, saliency, dorsal attention, visual, etc. network) [150]. To develop a new taxonomy of functions that does not refer to subjective constructs is a major endeavor for cognitive science.

A second question presents, we believe, a challenge to critics of functional modularity. As we have shown, the dependence of response time with rotational angle and its correlation with actual rotational movement are well-established characteristics of mental rotation. Simulation theories build on this or similar observations relating imagined and performed movement. The question we would like to ask is: why does there have to be such a dependence? Why is cognition not free to perform the task differently, for example by ‘rotating’ faster with larger rotational angles instead of maintaining the same speed? Additionally, why is this pattern of response so similar across different participants? The answers appear to be obvious: because cognition *cannot do otherwise* and because all people *do the task in a similar way*. In other words, mental rotation has at least some characteristics that would be ascribed to a functionally modular system: it performs the task automatically, always in the same way, produces always the same output and is encapsulated and universal. This observation suggests that some form of modularity must be present in complex systems such as the brain, even if encapsulation of information is a relative rather than an absolute characteristic.

A final point relates to the methodological approaches used to study neural reuse. For the last 30 years cognitive neuroscience has seen a strong shift towards functional neuroimaging as the prevailing paradigm of study. This dominance of one paradigm is accompanied with a tendency to disregard some shortcomings of the method, such as the sluggishness of the blood-oxygenation response, or the difficulty to decide whether the link between activation of a brain region and its underlying function is causal or epiphenomenal. We believe that a combination of approaches provides an opportunity to compensate for such weaknesses and thus to substantiate the claims and principles of neural reuse made in this article.
